# Local immunotherapy with the RNA-based immune stimulator CV8102 induces substantial anti-tumor responses and enhances checkpoint inhibitor activity

**DOI:** 10.1007/s00262-022-03311-4

**Published:** 2022-11-02

**Authors:** Johannes Lutz, Michael Meister, Mohamed Habbeddine, Katja Fiedler, Aleksandra Kowalczyk, Regina Heidenreich

**Affiliations:** 1 Curevac AG, Tübingen, Germany; 2 Present Address: Boehringer Ingelheim Pharma & Co. KG, Biberach/Riss, Germany; 3 Present Address: Hengrui Europe Biosciences AG, Zurich, Switzerland

**Keywords:** Intratumoral, Immunotherapy, TLR-7/8 agonist, RIG-I agonist, Anti-PD-1 treatment

## Abstract

Immunotherapy has revolutionized cancer treatment in recent years. Although currently approved checkpoint inhibitors (CPIs) yield remarkable anti-tumoral responses in several cancer types, a substantial proportion of patients do not benefit from such therapies. Local activation of innate immune signaling pathways is a promising approach to overcome the immunosuppressive tumor microenvironment, induce anti-tumor immunity, and improve the efficacy of CPI therapies. Here, we assessed the mode of action and efficacy of the RNA-based innate immune stimulator CV8102 for local immunotherapy in preclinical models. Intratumoral (i.t.) administration of CV8102 activated innate immune responses in the tumor microenvironment and draining lymph nodes, resulting in a dose-dependent anti-tumoral response. Combining i.t. CV8102 with systemic anti-programmed death protein 1 (PD-1) treatment further enhanced anti-tumoral responses, inducing tumor infiltration and activation of CD8^+^ T cells. The resulting memory response prevented tumor growth in rechallenged animals and impaired the growth of non-injected distal tumors. Therefore, i.t. CV8102 delivery is a promising approach for local cancer immunotherapy, especially in combination with CPIs. Clinical testing of CV8102 is ongoing (NCT03291002).

**Supplementary Information:**

The online version contains supplementary material available at 10.1007/s00262-022-03311-4.

## Background

Harnessing the body’s immune system to fight cancer has become one of the most promising therapeutic approaches in oncology [1]. The use of checkpoint inhibitors (CPIs) targeting T cell co-inhibitory receptors like cytotoxic T lymphocyte associated protein 4 (CTLA-4) or programmed death protein 1 (PD-1), induced strong and durable anti-tumoral responses in several tumor types and improved clinical outcomes for patients with a wide range of cancers; however, a substantial proportion of patients remain unresponsive to CPIs, so a significant unmet need remains [2].

It has become apparent that the status of the tumor microenvironment is critical for the efficacy of CPI therapy [3]. Accordingly, strategies to overcome the immune suppression and induce local, pro-inflammatory responses within tumors have gained attention, and local activation of the innate immune system by triggering pattern recognition receptors such as toll-like receptors (TLRs) or retinoic acid-inducible gene I (RIG-I) have demonstrated great potential in this regard [4, 5].

TLRs are a class of pattern recognition receptors (PRRs) that can recognize pathogens and damage-associated molecular patterns, such as lipopolysaccharide and free nucleic acids [6]. TLR7 and TLR8 are present in the endosomal membrane, where they recognize pathogen-derived purine-rich single stranded RNA [7]. While TLR7 is primarily expressed in myeloid cells (dendritic cells [DCs], monocytes and macrophages) [8] and to a lesser degree in other leukocytes including natural killer (NK) cells, and T cells, TLR8 is predominately expressed in myeloid DCs, monocytes, macrophages, and T cells [9, 10]. Upon activation, both receptors signal through MyD88 and interleukin-1 receptor-associated kinase 4 to mediate production of type I interferons (IFNs) and other pro-inflammatory molecules [11, 12]. As well as being candidate vaccine adjuvants [13], synthetic TLR7/8 agonists have potent anti-tumor activity when used alone or in combination with immunotherapies [10]. To limit the systemic exposure, which causes severe side effects and toxicity, TLR7/8 agonists are predominantly employed locally [13, 14]. Topical application of imiquimod (TLR7 agonist) or resiquimod (TLR7/8 agonist) has been used successfully to treat several murine tumor models [15–17], while resiquimod has also demonstrated efficacy in early-stage cutaneous T-cell lymphoma [18]. Imiquimod is also approved as a topical treatment for actinic keratosis and superficial basal cell carcinoma [19, 20]. Furthermore, combining CPIs with application of intratumoral TLR7/8 activation results in myeloid cell activation and antigen-presenting cell (APC) maturation in the tumor microenvironment to augment the activity and specificity of adaptive immune responses [21].

The cytosolic RNA receptor RIG-I has also been linked to anti-tumor immune responses [22, 23]. RIG-I is expressed in most nucleated cells including tumor cells [24] and recognizes various single-stranded and double-stranded RNAs, preferentially with 5’ triphosphate ends, or polyuridine (poly-U) rich sequences, following events such as viral or bacterial infection [25, 26]. In myeloid cells, RIG-I activation induces pro-inflammatory cytokines and type-I IFN production as well as inflammasome activation [23, 27], resulting in broad innate and adaptive immune responses. In tumor cells, RIG-I activation has been shown to induce immunogenic cell death, which promotes cross-presentation of tumor-associated antigens through bystander DCs and augments the efficacy of CTLA-4 checkpoint blockade [27–29].

In previous studies, we demonstrated that synthetic RNA molecules can activate innate immunity by triggering intracellular PRRs [30]. Following this discovery, we developed the potent, RNA-based, immuno-stimulatory agent CV8102 which comprises a single-stranded, non-coding, non-capped RNA with poly-U repeats, complexed by a cationic peptide. CV8102 mediates its immunostimulatory properties by simultaneously triggering TLR7/8 and RIG-I signaling [31]. In preclinical models, intradermal, intramuscular, and intravenous application of CV8102 showed a strong adjuvant function together with good tolerability and a favorable safety profile in settings of therapeutic cancer vaccines and in prophylactic vaccines for infectious diseases [31, 32]. CV8102 induced locally restricted, transient but strong up-regulation of pro-inflammatory and anti-viral cytokines including type-I IFNs and cytoplasmic RNA sensors. This was followed by the activation of DCs, NK cells, B cells and T cells, effectively boosting humoral and cellular responses against vaccine antigens [31, 32]. CV8102 also activated human peripheral blood mononuclear cells (PBMCs) by inducing the expression of a plethora of cytokines and chemokines including type-I IFNs leading to the activation and maturation of DCs [32–34]. In a clinical setting, intramuscular CV8102 enhanced the immunogenicity of a licensed rabies vaccine in a first-time-in-human (FTiH) trial [35] and was included as an intradermal adjuvant in the HepaVac-101 FTiH therapeutic cancer vaccine that was assessed in a phase I/II clinical trial in patients with hepatocellular carcinoma [36].

In the present study, we evaluated the potential of i.t. administration of CV8102, with and without systemic anti-PD-1 therapy, to modulate the tumor microenvironment and to induce anti-tumoral immune responses in mouse tumor models.

## Methods

### CV8102

CV8102 consists of a synthetic RNA and a polymeric carrier. The RNA component consists of an uncapped, U-rich RNA sequence containing several poly-U-repeats as described in WO2009/095226. The RNA component is complexed with a polymeric carrier formed by a disulfide-crosslinked cationic peptide (WO2012013326).

Dephosphorylated CV8102 was produced using RNA treated with calf intestinal alkaline phosphatase. 5’ ends of the RNA were cleaved off using a chemical scissor and separated by ion-pair reversed-phase high-performance liquid chromatography to measure dephosphorylation efficacy.

### Mice

Female BALB/c mice (aged 7–9 weeks) were obtained from Janvier Laboratories (France) and kept under specific pathogen-free conditions. The animal experimental protocols were approved by the ethics committee of the Tübingen Regional Council.

### In vitro cell stimulation

CT26 colon carcinoma tumor cells (ATCC) were grown in RPMI-1640 with 1% L-glutamine, 1% Penicillin–Streptomycin, and 10% heat inactivated fetal calf serum. For stimulation, cells were seeded at 100,000 cells/mL in 6-well plates and were on the following day either incubated with 100 µg/mL of CV8102, dephosphorylated CV8102, low molecular weight Poly(I:C) (average size 0.2–1 kb, Invivogen), or high molecular weight Poly(I:C) (average size 1.5–8 kb, Invivogen), or transfected with 1 µg/mL CV8102 or dephosphorylated CV8102, formulated in Lipofectamine 2000 (Invitrogen).

### Tumor challenge and treatment

BALB/c mice were challenged subcutaneously (s.c.) on one or both flanks with 1 × 10^6^ CT26 cells or 0.5 × 10^6^ A20 murine B cell lymphoma cells (ATCC) per flank. One day prior to first treatment, mice were grouped based on equal tumor size distribution. If mice were challenged on both flanks, CT26 tumors cells were implanted (s.c.) 5 days apart. For all challenge experiments, the day of the first challenge was considered Day 0.

Following challenge, mice were treated as indicated in the individual figure legends with CV8102 resuspended in Ringer’s lactate buffer (25–100 µg, i.t.), rat immunoglobulin G (IgG) 2a anti-mouse PD-1 antibody (200 µg, intraperitoneal (i.p.) [CD279, clone RMP1-14; BioXCell]) in phosphate buffered saline, or both in combination. Ringer’s lactate buffer (i.t.) served as the control for CV8102 treatment, and rat IgG2a isotype (i.p.) served as the control for anti-PD-1 treatment.

Tumor growth was measured using calipers and tumor volume was calculated as: length × width^2^ × π/6. Animals that had a non-measurable tumor at the end of the study were scored as complete responders. Re-challenge of CT26 complete responders was performed four months after the initial challenge by implanting 1 × 10^6^ CT26 cells (s.c.) into the opposite flank.

Survival (challenged animals remaining in the study) was defined as not reaching a humane endpoint and a tumor volume of < 2000 mm^3^ (CT26 cell challenge on a single flank) or < 1000 mm^3^ (A20 cell challenge on a single flank or CT26 cell challenge on both flanks).

### Cytokine measurements

Supernatants of CT26 cells stimulated in vitro were measured using the LEGENDplex™ Multi-Analyte Flow Assay Kit (Mouse Anti-Virus Response Panel, BioLegend). CT26 tumors treated in vivo were snap-frozen and lyzed in a TissueLyzer (Qiagen) using T-Per tissue protein extraction solution (Thermo Scientific) and a protease inhibitor cocktail (cOmplete Mini, Roche). Lysates were adjusted for protein content (150 µg) and analyzed with the Cytometric Bead Assay Kit (BD Biosciences) for CCL2 (MCP1), CCL3 (MIP-1α), CCL4 (MIP-1β), CCL5 (RANTES), CXCL1 (KC), CXCL9 (MIG), GM-CSF, IFN-γ, IL-1α, IL-1β, IL-2, IL-4, IL-6, IL-10, IL-12p70, TNF. Mouse CXCL10 (IP-10) concentrations were measured by DuoSet ELISA (R&D systems).

### Flow cytometry

In vitro stimulated CT26 cells were stained with Fixable Aqua Dead Cell Stain (ThermoFisher) and antibodies against MHC-I (H-2Kd/H-2Dd, Invitrogen) and PD-L1 (Biolegend). CT26 tumors treated in vivo were harvested into MACS® Tissue Storage Solution and processed into single cell suspensions using the Tumor Dissociation Kit and a gentleMACS™ Dissociator (all Miltenyi Biotec). Cells were stained with Fixable Aqua Dead Cell Stain, incubated with Fc-Block and surface staining antibodies (see Table S1). Cells were then either fixed with BD Cytofix™ Fixation Buffer (BD Biosciences) or permeabilized with Foxp3 Fixation/Permeabilization working solution (eBioscience) before staining intracellularly (see Table S1). Cells were analyzed on a LSR Fortessa using FlowJo software (BD Biosciences).

### RNA-Seq analysis

BALB/c mice were challenged s.c. with 1 × 10^6^ CT26 cells on the flank. At days 10 and 14 after tumor challenge, mice were treated with 100 µg CV8102 (i.t.) or 200 µg of anti-PD-1 antibody (i.p.), either alone or in combination. Control animals received Ringer’s lactate buffer (i.t.). Tumors were collected 5 h after the second treatment and snap frozen. Total RNA was extracted and used to generate stranded, poly-A enriched RNA TruSeq libraries (Illumina) that were sequenced on Illumina NextSeq, v2, 1 × 75 bp (target 30 million reads per sample). Differential gene expression analysis was performed using reference genome mm10. Gene sets analysis was performed using Kyoto Encyclopedia of Genes and Genomes (KEGG) pathway or Gene Ontology (GO) functional clustering. GO enrichment analysis of genes upregulated ≥ 1.5-fold was performed using http://geneontology.org. Shown GO terms include: defense response to virus (GO:0051607), cellular response to IFN-α (GO:0035457), cellular response to IFN-β (GO:0035458), positive regulation of RIG-I (GO:1900246), and regulation of ribonuclease activation (GO:0060700).

### Statistical analyses

Statistical analyses were performed using GraphPad Prism software, Version 9.3. Details of each analysis are provided in the associated figure legend; p < 0.05 was considered statistically significant.

## Results

### CV8102 induces type-I IFN production in tumor cells in vitro

CV8102 has been shown to directly stimulate human PBMCs, inducing a type-I IFN response and expression of various cytokines and chemokines including TNF, IL-6, IL-1β, and chemokine (C-X-C motif) ligand (CXCL) 9/10 [32–34]. To determine whether CV8102 could also act directly on tumor cells to induce cytokine secretion, we stimulated murine CT26 colon carcinoma tumor cells in vitro with CV8102 by adding it directly into the culture media or by transfecting it into the cells using a lipofection agent.

**Fig. 1:**
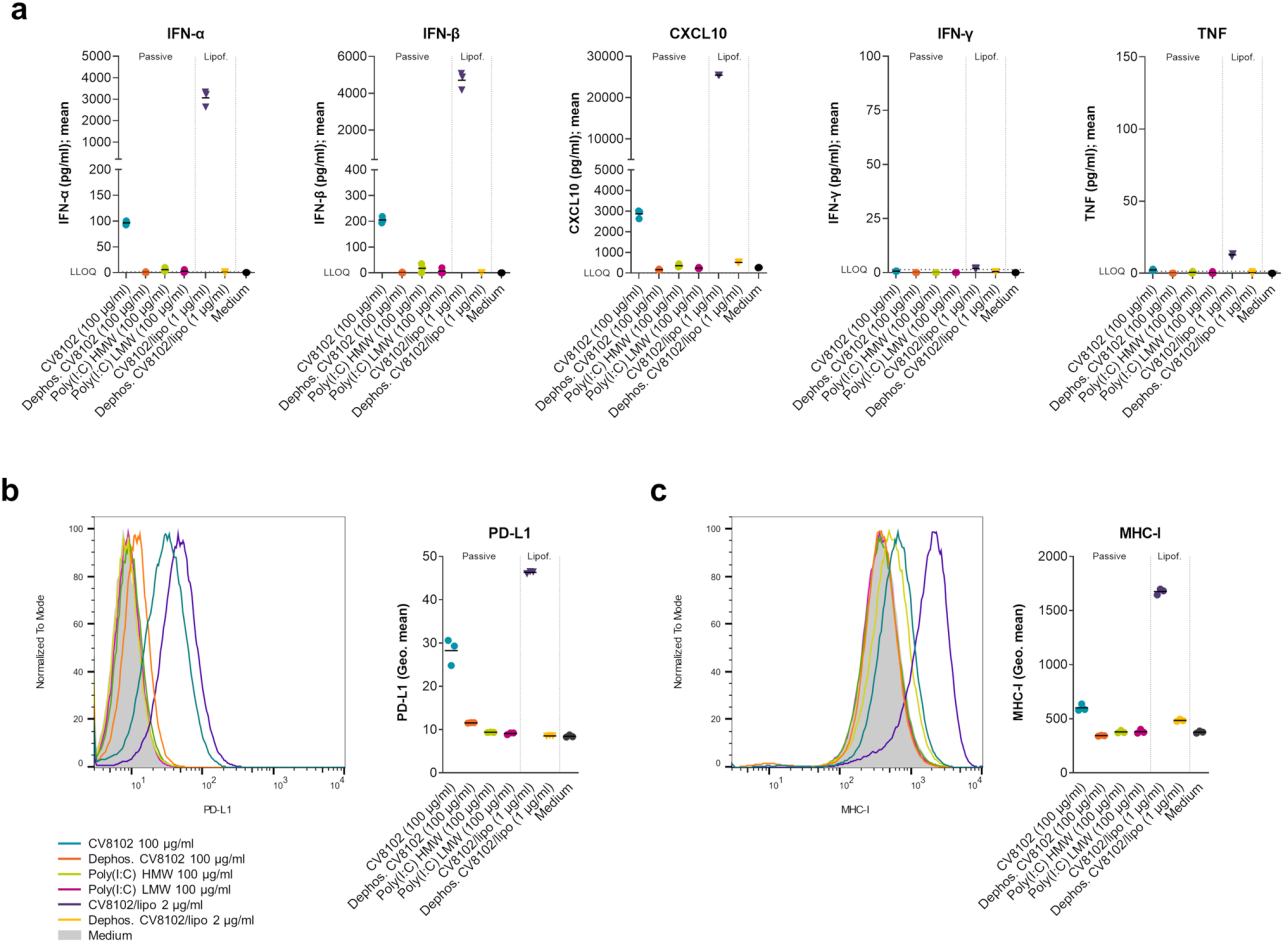
CV8102 induces cytokine release and upregulation of PD-L1 and MHC-I on tumor cells in vitro. **a**–**c** CT26 cells were incubated with 100 µg/ml CV8102, dephosphorylated CV8102, and high or low molecular weight (HMW, LMW) poly(I:C) or transfected with 1 µg/ml CV8102 and dephosphorylated CV8102. After 22 h, cytokines in the supernatant **a** and surface expression of PD-L1 **b** and MHC-I **c** on the cells were measured. Horizontal dotted lines represent the lower limit of quantification (LLOQ) in (**a**)

Analysis of supernatants demonstrated that stimulation with CV8102, which contains a 5’-triphosphate group (5’ppp), induced the secretion of IFN-α, IFN-β, CXCL1, CXCL10, chemokine (C–C motif) ligand (CCL) 2, CCL5, IL-1β, IL-6, IL-10, and GM-CSF, but only negligible amounts of IFN-γ or TNF (Fig. 1a, Fig. S1). This effect was more pronounced when CV8102 was delivered using lipofection, compared with passive incubation in the culture media. In contrast, stimulation with dephosphorylated CV8102 or Poly(I:C), which both lack 5’ppp, failed to induce these cytokines and chemokines, suggesting the involvement of RIG-I.

On a cellular level, stimulation with CV8102 led to an upregulation of PD-L1 and MHC-I on CT26 cells (Fig. 1b,c). Again, this was not observed following stimulation with dephosphorylated CV8102 or Poly(I:C).

### Intratumoral CV8102 administration induces dose-dependent anti-tumoral effects

**Fig. 2:**
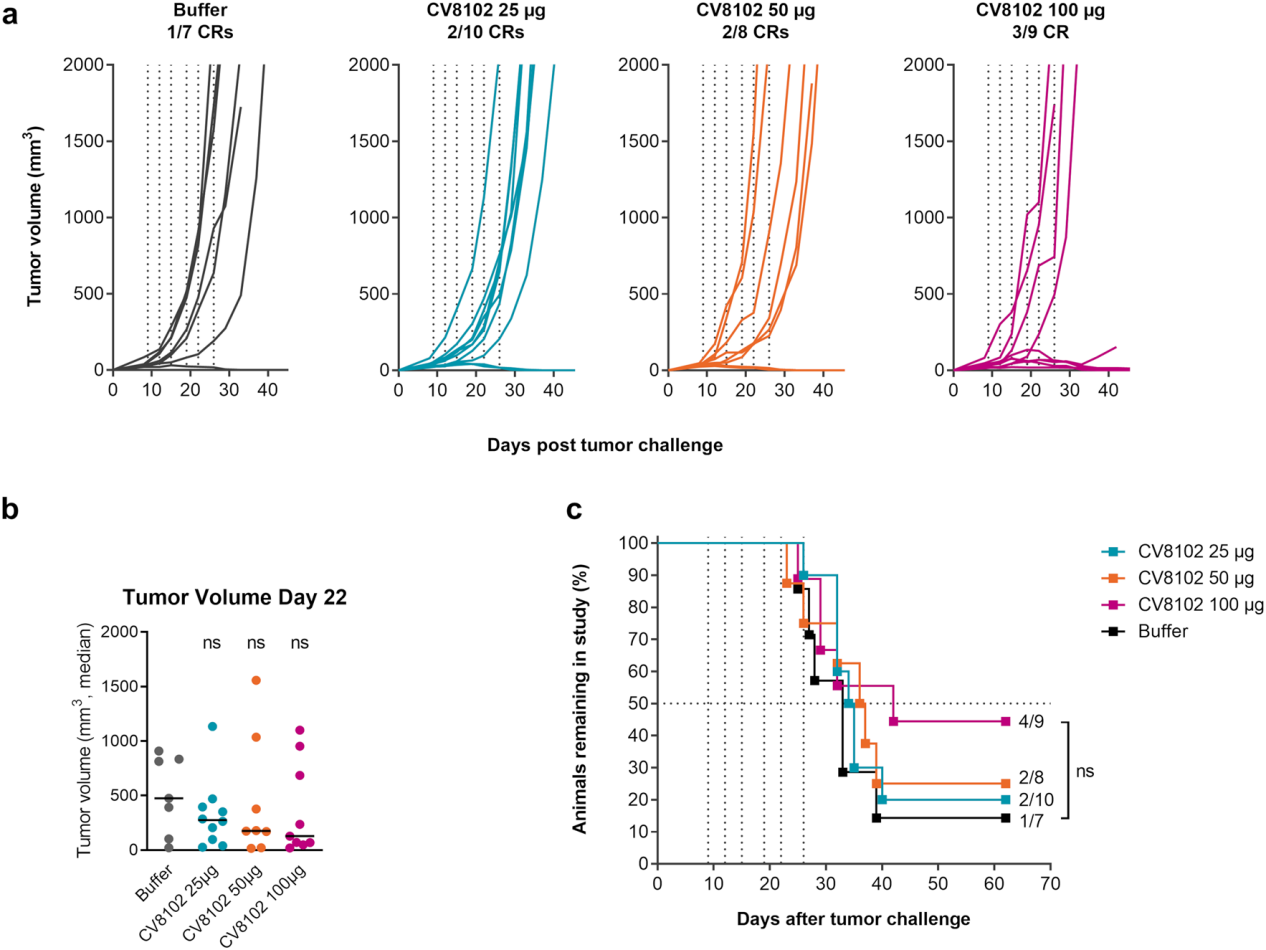
Intratumoral administration of CV8102 induces dose-dependent tumor regression in CT26 tumors. **a**–**c** Mice (n = 7–10/group) were challenged on one flank with CT26 tumor cells on Day 0 and treated on Days 9, 12, 15, 19, 22, and 26 with 25 µg, 50 µg, or 100 µg CV8102 (i.t.). **a** Tumor volume over time for individual mice. Complete responders (CRs) and group sizes are indicated. **b** Tumor volume at Day 22 after tumor challenge. **c** Survival using tumor volume cut-off of 2000 mm^3^. Animals remaining in study and group sizes are indicated. Statistical analysis by Mann–Whitney test of treatments versus buffer (**b**) and Mantel-Cox test (**c**). ns = not significant. Treatment days are indicated as vertical dotted lines

To evaluate the anti-tumoral potential of CV8102 in vivo, BALB/c mice were challenged s.c. with CT26 cells, and treated twice weekly with 25, 50, or 100 µg CV8102 (i.t.) for three weeks. In animals where the CT26 tumors became established, the trajectory of tumor volume increase was similar; however, the proportion of animals in which tumors failed to grow increased with CV8102 dose, with the effect most pronounced in the 100 µg group (Fig. 2a). The number of complete responders was 1/7 for buffer-treated mice compared with 3/9 for CV8102 treated mice at the highest dose. Tumor volume at Day 22 and survival showed a similar trend of improvement at the highest CV8102 dose; however, the results were not statistically significant compared with buffer-treated animals (Fig. 2b,c).

**Fig. 3:**
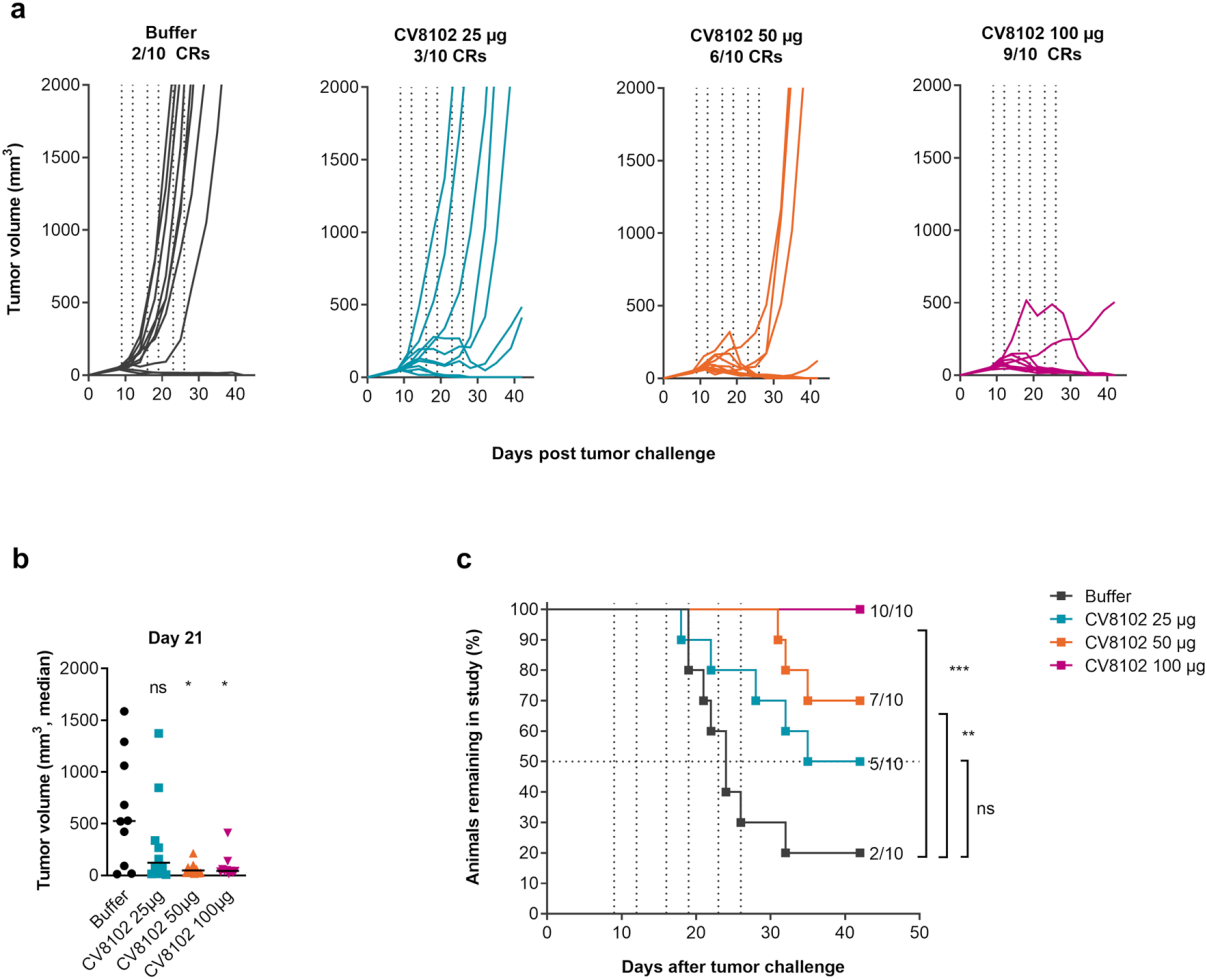
Intratumoral administration of CV8102 induces dose-dependent tumor regression in A20 tumors. Mice (n = 10/group) were challenged on one flank with A20 tumor cells and treated on Days 9, 12, 16, 19, 23, and 26 with 25 µg, 50 µg, or 100 µg CV8102 (i.t.). **a** Tumor volume over time for individual mice. Complete responders (CRs) and group sizes are indicated. **b** Tumor volume at Day 21 after tumor challenge. **c** Survival using tumor volume cut-off of 1000 mm^3^. Animals remaining in study and group sizes are indicated. Statistical analysis by Mann–Whitney test of treatments versus buffer (**b**) and Mantel-Cox test (**c**). *p < 0.05, **p < 0.01, ***p = 0.003, ns = not significant. Treatment days are indicated as vertical dotted lines

The B cell lymphoma cell line A20 was used as a second murine tumor model, and a similar treatment schedule was applied. Compared with the CT26 tumor model, a more notable dose-dependent suppression of tumor growth was observed. While the 25 µg dose of CV8102 caused tumor growth delay in a subset of mice compared with the buffer-treated controls, 50 µg CV8102 initiated complete tumor rejection in 60% of mice. Almost complete tumor eradication and significantly enhanced overall survival was observed with 100 µg CV8102 (Fig. 3a–c).

### Synergy between intratumoral CV8102 treatment and systemic anti-PD-1 treatment

As the suppressive immune regulatory molecule PD-L1 was upregulated on CT26 cells in vitro after stimulation with CV8102 (Fig. 1b), we speculated this could suppress tumor-infiltrating lymphocytes and thereby partially counteract the immune stimulatory effects of the CV8102-induced cytokines. Accordingly, locally administered CV8102 (100 µg, i.t.) was combined with systemic anti-PD-1 antibody treatment (200 µg, i.p.) to prevent this inhibition.

**Fig. 4:**
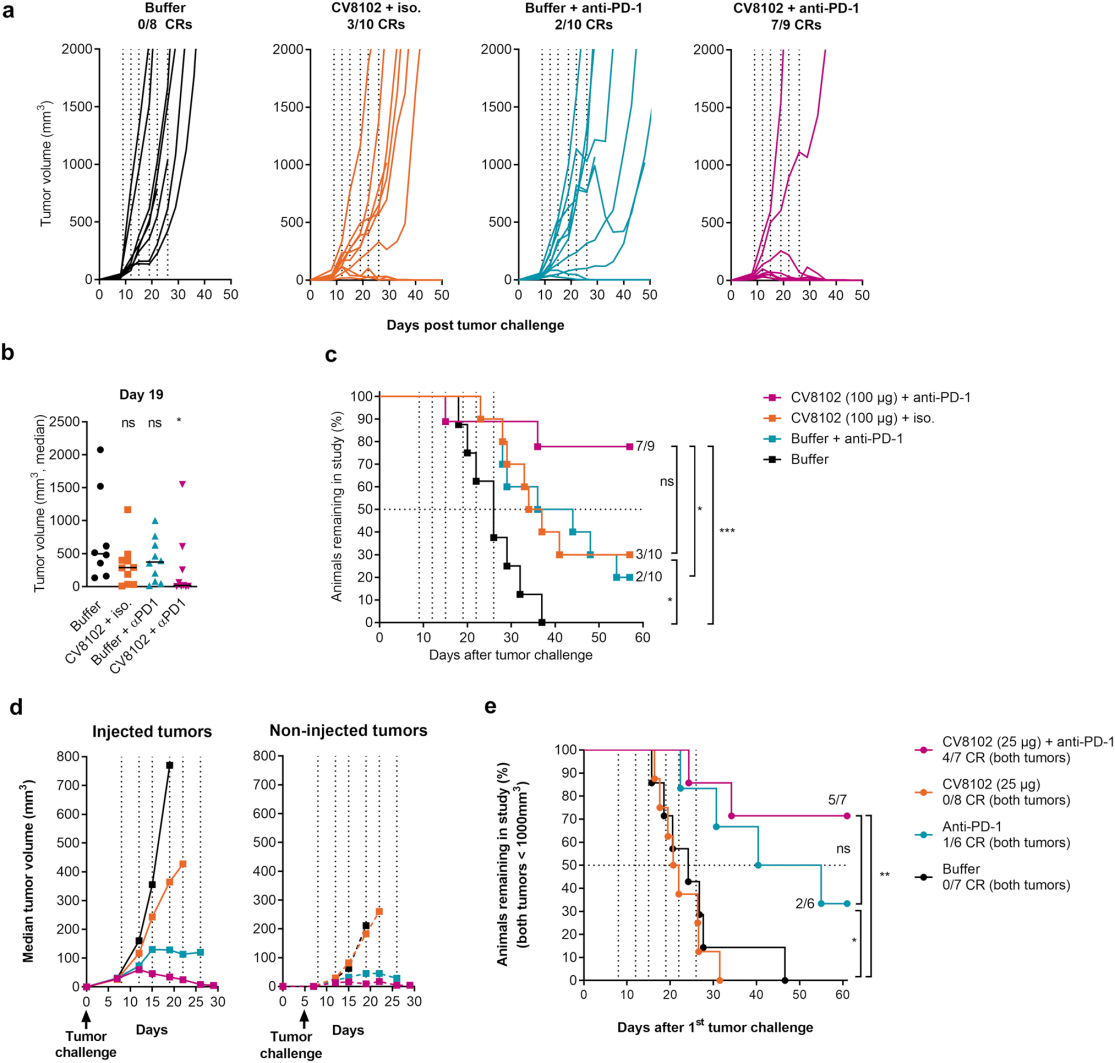
Intratumoral application of CV8102 boosts the anti-tumor effect of systemic anti-PD-1 treatment. **a**–**c** Mice (n = 8–10/group) were challenged on one flank with CT26 tumor cells on Day 0 and treated on Days 9, 12, 15, 19, 22, and 26 with CV8102 (100 µg, i.t.) or buffer (i.t.), and with anti-mouse PD-1 antibody or isotype control (200 µg, i.p.). **a** Tumor volume over time for individual mice. Complete responders (CRs) and group sizes are indicated. **b** Tumor volume at Day 19 after tumor challenge. **c** Survival using tumor volume of 2000 mm^3^ as cut-off. Animals remaining in study and group sizes are indicated. **d**, **e** Mice (n = 6–8/group) were challenged with CT26 tumor cells on Day 0 on the right flank and on Day 5 on the left flank. On Days 8, 12, 15, 19, 22, 26 mice received anti-mouse PD-1 antibody (200 µg; i.p.) and the tumor on the right flank was treated with CV8102 (25 µg, i.t.) or buffer (i.t.). The tumor on the left flank was not directly treated. **d** Median tumor volume for i.t. treated tumors and non-intratumorally treated tumors over time for as long as each treatment group remained complete. **e** Survival using tumor volume of 1000 mm^3^ for each of the tumors as cut-off. Animals remaining in study and group sizes are indicated in the graph. Complete responders (CR), i.e., mice without measurable tumors in both the right (injected) and left (non-injected) flanks, are indicated in the legend. Statistical analysis by Mann–Whitney test of treatments versus buffer (**b**) and Mantel-Cox test (**c, e**). *p < 0.05, *p = 0.0242, ***p = 0.001, ns = not significant. Treatment days are indicated as vertical dotted lines

While treatment with CV8102 plus isotype control antibodies, or buffer (i.t.) plus anti-PD-1 antibodies were each associated with modest reductions in tumor growth and improved survival, combining both treatments resulted in significantly delayed tumor growth and significantly improved overall survival compared with buffer-treated control mice, and mice treated with anti-PD-1 antibodies alone (Fig. 4a–c). The increase in overall survival seen with combined CV8102 and anti-PD-1 treatment was accompanied by a 77% complete response rate. When mice that had cleared the tumor were re-challenged four months after the primary challenge with the same tumor and left untreated, no tumor growth was observed, demonstrating initial treatment had established sufficient immunological memory to eradicate the re-challenge (Fig. S2).

### Combination of intratumoral CV8102 plus anti-PD-1 antibodies can eradicate non-injected distant tumors

To investigate whether the anti-tumor effects of CV8102 with or without systemic anti-PD-1 treatment could extend its reach beyond the locally treated primary tumor, mice were challenged on both flanks with CT26 tumor cells five days apart. The primary tumor (implanted first; Day 0) was treated with CV8102 (25 µg) or buffer, whilst the secondary tumor (implanted on Day 5) received no local CV8102 or buffer treatment. In addition, some mice received systemic anti-PD-1 antibody treatment (200 µg).

Anti-PD-1 antibodies alone reduced the growth of both tumors, resulting in 1/6 complete responders and an increased survival rate compared with buffer-treated animals. In keeping with observations from the single tumor CT26 model (Fig. 2), treatment with 25 µg CV8102 alone had no notable effect on injected or non-injected tumor growth; however, the combination with systemic anti-PD-1 antibodies led to stronger growth inhibition of both tumors than anti-PD-1 antibodies alone, resulting in 4/7 complete responders, and a 71% survival rate (Fig. 4d,e).

### Intratumoral CV8102 induces an innate immune response, which is enhanced when combined with systemic anti-PD-1 treatment

To assess the mechanisms by which CV8102 mediates its anti-tumoral activity and boosts the anti-tumoral effects of anti-PD-1, RNA-Seq analysis of the CT26 tumors, which included the tumor cells, stromal cells and immune cells was performed 5 h after the second treatment with 100 µg CV8102, 200 µg anti-PD-1 antibody, or both treatments combined.

**Fig. 5:**
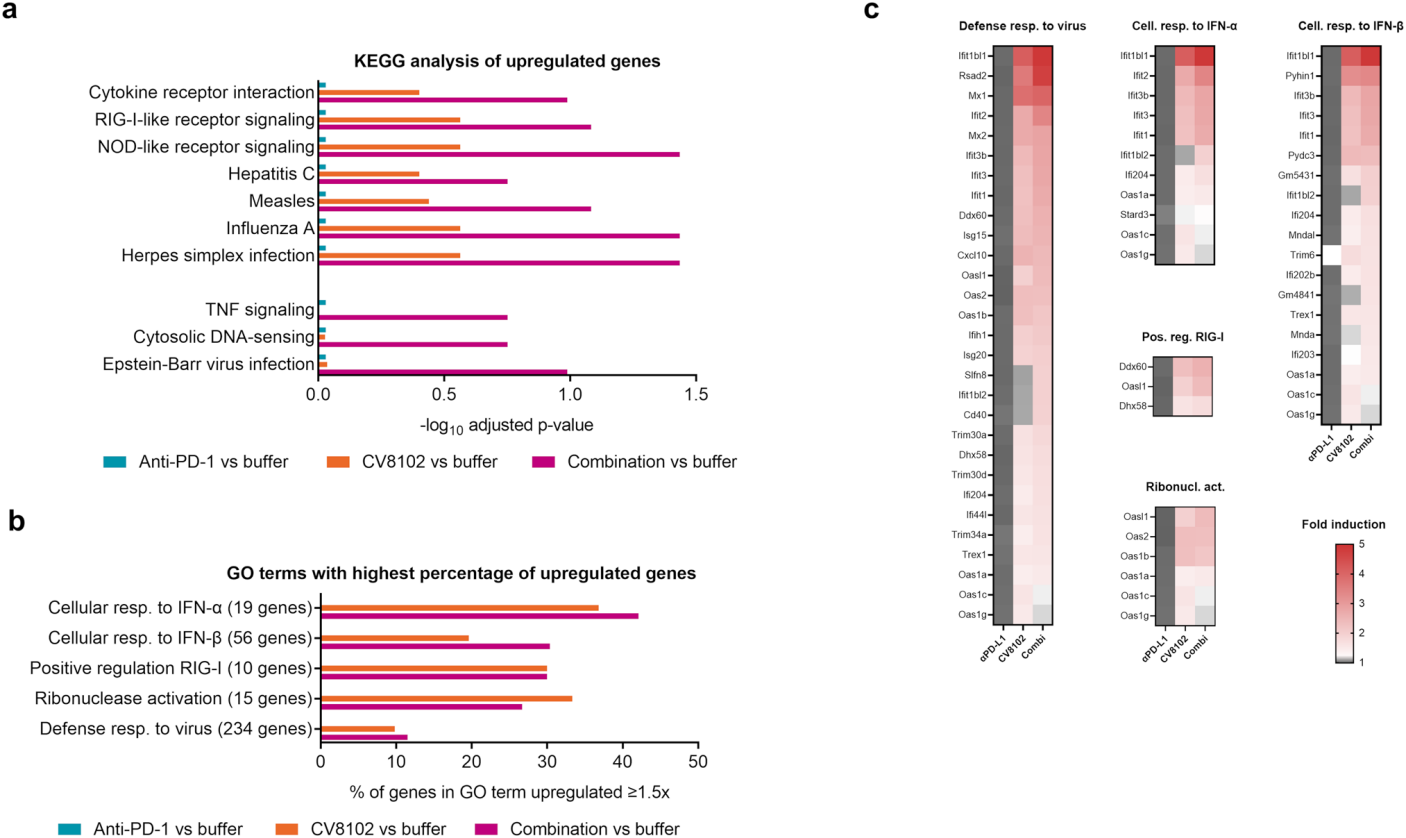
Early activation of anti-viral immunity in the tumor microenvironment following intratumoral administration of CV8102, with and without systemic anti-PD-1 treatment. Mice (n = 6/group) were challenged on one flank with CT26 tumor cells on Day 0 and treated on Days 10 and 14 with 100 µg CV8102 (i.t.) and 200 µg anti-PD-1 antibodies (i.p.) either alone or in combination; control animals received buffer (i.t.). Tumors were collected 5 h after the second treatment and analyzed by RNA-Seq. **a** Enriched gene sets in individual or combination treatments compared with buffer treatment by KEGG pathway analysis. Adjusted p-values are shown. **b**, **c** Genes with ≥ 1.5-fold increase in expression for one of the treatments compared with buffer treatment were clustered by GO biological functions. **b** GO terms with highest coverage of genes upregulated ≥ 1.5-fold: Defense response to virus, cellular response to IFN-α, cellular response to IFN-β, positive regulation of RIG-I, regulation of ribonuclease activation. Number of genes contained in each GO term (in brackets) and percentage of genes upregulated ≥ 1.5-fold are shown. **c** List of all genes upregulated ≥ 1.5-fold for at least one treatment in the selected GO terms

A KEGG pathway analysis of upregulated transcripts showed enrichments of gene sets associated with cytokines, RIG-I-like and nucleotide-binding and oligomerization domain (NOD)-like receptor signaling, and viral infections in CT26 tumors treated with CV8102 compared with buffer-treated tumors (Fig. 5a). Combination treatment with CV8102 and anti-PD-1 antibodies increased the upregulation of these gene sets and induced additional gene sets associated with TNF signaling, cytosolic-DNA-sensing and Epstein-Barr virus infection, indicating a synergistic effect of anti-PD-1 treatment with CV8102. In contrast, treatment with anti-PD-1 antibodies alone was not associated with any enrichment of these gene sets.

A GO analysis of biological processes for transcripts upregulated at least 1.5-fold compared with buffer controls yielded for CV8102 and combination-treated tumors immune-related terms such as “*defense responses to viruses*” (Fig. 5b). Coverage was especially high for the terms “*cellular responses to IFN-α*”, “*cellular responses to IFN-β*”, “*positive regulation of RIG-I”*, and “*ribonuclease activation”*, whereby combined treatment was associated with the ≥ 1.5-fold upregulation of 8/19, 17/56, 3/10, and 4/15 term members, respectively. As with the KEGG analysis, treatment with anti-PD-1 antibodies alone was not associated with any enrichment of these or other gene sets. Many of the upregulated genes belong to the *interferon-induced protein with tetratricopeptide repeats* (Ifit) and *2'-5' oligoadenylate synthetase* (Oas) protein families, which block translation or cause degradation of viral RNA, respectively (Fig. 5c). Further prominent transcripts are the key anti-viral protein *radical S-adenosyl methionine domain-containing protein 2* (Rsad2) [37], CXCL10, and CD40.

### Synergy of intratumoral CV8102 and systemic anti-PD-1 treatment enables adaptive immune system activation

**Fig. 6:**
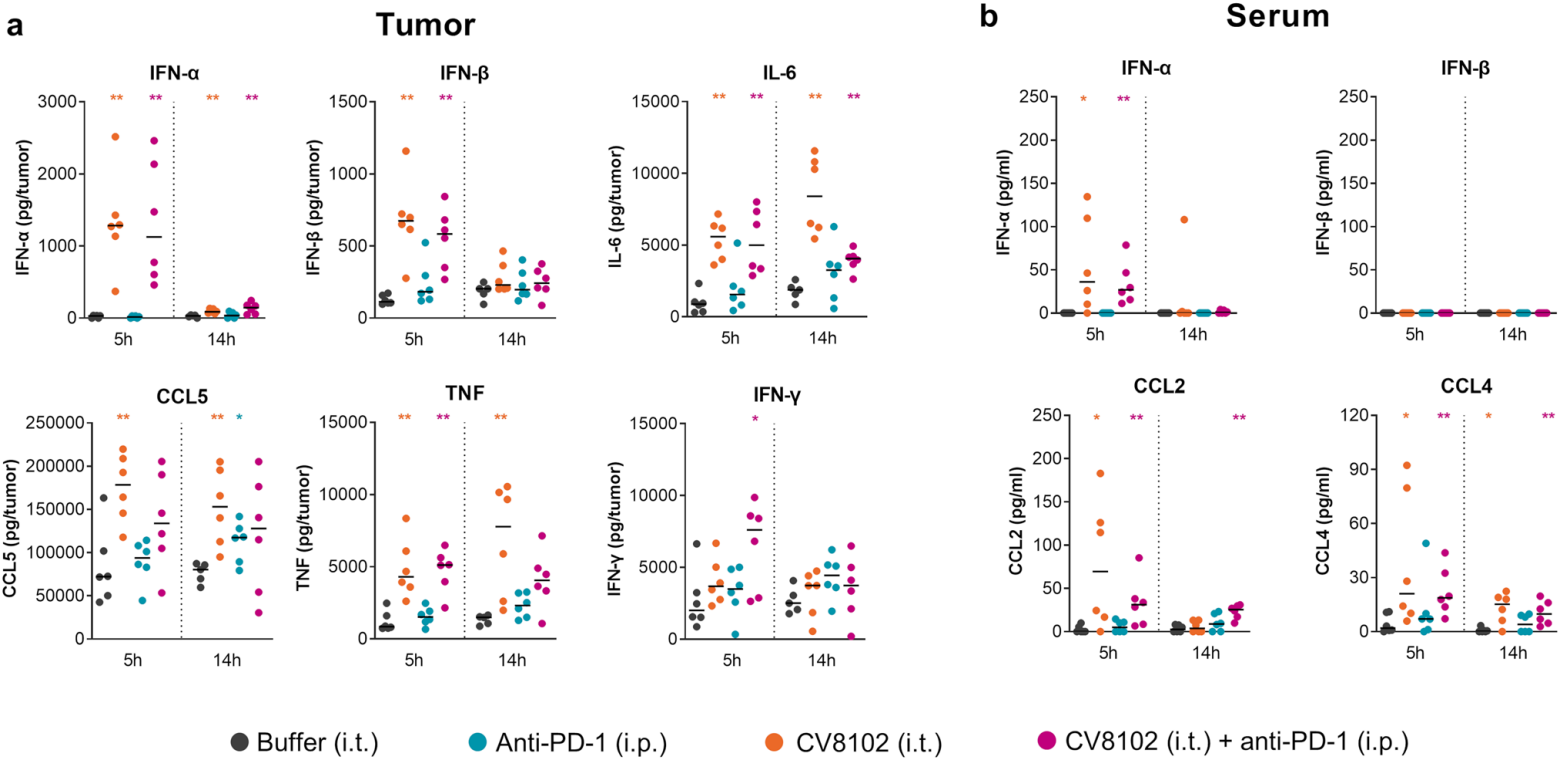
Intratumoral application of CV8102 induces cytokine release. Mice (n = 6/group) were challenged on one flank with CT26 tumor cells on Day 0 and treated once on Day 14 with 100 µg CV8102 (i.t.) and 200 µg anti-PD-1 antibodies (i.p.) either alone or in combination; control animals received buffer (i.t.). Tumors and sera were collected 5 h and 14 h later, and cytokine concentrations were determined in tumor lysates (**a**) and sera (**b**). Statistical analysis by Mann–Whitney test. All treatments are compared with buffer controls and significant changes are shown (*p < 0.05, **p < 0.01)

To build on the RNA-Seq results and assess the direct treatment effects, cytokine concentrations were analyzed in the tumor and serum at 5, 14, 24 and 72 h after a single treatment, 14 days after CT26 challenge. Within the tumor, CV8102 alone, and in combination with systemic anti-PD-1 treatment, induced similar amounts of IFN-α, IFN-β, IL-6, and TNF, while anti-PD-1 antibodies alone did not induce these cytokines (Fig. 6a). The induction was short-lived and returned to baseline after 14 h for IFN-α and IFN-β, and after 72 h for IFN-γ, IL-6, TNF and CCL5 (data not shown). The increased IFN-α concentrations in the tumor led to a transient increase in serum IFN-α concentrations (Fig. 6b). Similar results for the tumor and serum were also obtained after three injections, indicating that effects are maintained after repeated treatment (Fig. S3).

**Fig. 7:**
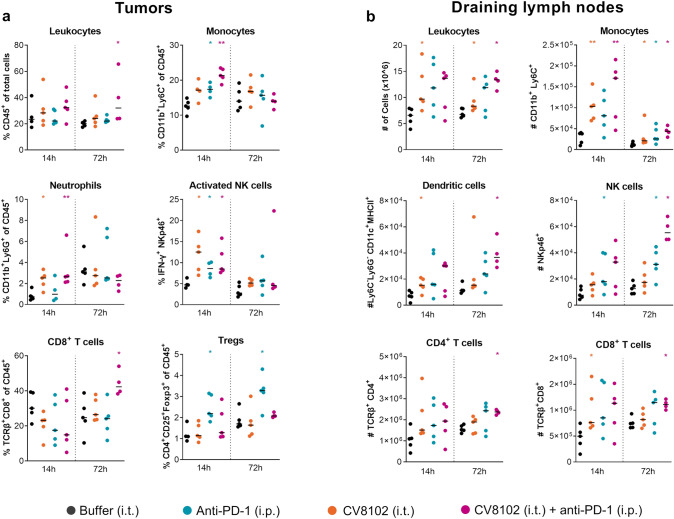
Concomitant intratumoral CV8102 and systemic anti-PD-1 treatment induces lymphocyte infiltration and activation. Mice (n = 5/group) were challenged on one flank with CT26 tumor cells on Day 0 and treated on Days 9 and 13 with 100 µg CV8102 (i.t.) and 200 µg anti-PD-1 antibodies (i.p.) either alone or in combination; control animals received buffer (i.t.). Tumors **c** and draining lymph nodes **d** were collected 14 h or 72 h after the second treatment, and cellular composition was analyzed by flow cytometry. Statistical analysis by Mann–Whitney test. All treatments are compared with buffer controls and significant changes are shown (*p < 0.05, **p < 0.01)

Immune cell composition in tumors and draining lymph nodes (dLNs) were analyzed 14 and 72 h after the second treatment (Days 9 and 13), as these timepoints correspond with observations of tumor regression in previous experiments (Fig. 4a). In the tumor, CV8102 induced significant changes within the myeloid cell compartment early after treatment (Fig. 7a). These were most pronounced in the CV8102 plus anti-PD-1 treatment group, which was associated with increased frequencies of monocytes, neutrophils, and activated NK cells in the tumors 14 h after the second treatment, compared with the buffer-treated group. Frequencies of regulatory T cells (Tregs) were increased at 14 and 72 h after the second treatment with anti-PD-1 antibodies alone. Interestingly, this effect was absent in groups treated with the combination of anti-PD-1 antibodies and CV8102 or CV8102 alone. (Fig. 7a).

In dLNs, combined CV8102 and anti-PD-1 treatment led to an increase in the number of monocytes and NK cells at 14 h, and leukocytes and NK cells at 72 h after the second treatment (Fig. 7b). Additionally, an increase in DCs and CD4^+^ and CD8^+^ T cells was observed in the combined treatment group at 72 h after the second treatment.

## Discussion

Anti-tumoral immune responses are often inhibited by an immunosuppressive or immune-exclusive tumor microenvironment. Here, we demonstrated that i.t. injection of CV8102 can modulate the tumor microenvironment, increase immune activation, and improve anti-tumor responses; an effect that was enhanced by concomitant systemic anti-PD-1 antibody treatment.

Previously, we demonstrated that CV8102 stimulates TLR-7/8 and RIG-I and activates PBMCs [31, 32]. Recent studies exploring the properties of CV8102 on PBMCs from liver cancer patients and patients undergoing chemotherapy demonstrated strong immune-stimulatory effects, suggesting CV8102 may also be efficacious in cancer patients with severe immune impairment [33, 34]. Here, we demonstrated that CV8102 also acted directly on tumor cells in vitro to induce cytokine and chemokine secretion, leading to increased MHC-I expression on tumor cells, which may support presentation of tumor antigens to the immune system. The effect is dependent on the 5’ppp of CV8102 and is likely mediated by the 5’ppp receptor RIG-I. This has the advantage that RIG-I is expressed by most cells in the human body, including tumor cells [38], while other PRRs are primarily restricted to immune cell subsets. For example, TLR7, which senses single-stranded RNA and is also triggered by CV8102, is primarily expressed in plasmacytoid dendritic cells, monocytes, and B cells [39]. RIG-I-like receptor (RLR) activation induces immunogenic cell death, accompanied by increased inflammatory cytokine production, antigen presentation, and immunity against tumor antigens [40].

The in vivo activity of CV8102 when administered as monotherapy to both A20 and CT26 murine tumor models, led to reductions in tumor volume, coupled with increases in both survival rates and numbers of mice who were complete responders. In the A20 lymphoma model, there was a significant, dose-dependent effect on tumor growth, survival, and complete response rate. Although the same trend was apparent in the CT26 colon carcinoma model, the treatment effects were less pronounced. This distinction likely reflects differences in PRR expression between the tumor cells, their capacity to present antigens, as well as differences in the tumor microenvironment. However, the higher sensitivity of the A20 tumors to CV8102 treatment is unexpected as they are immunologically cold and dominated by leukemic B cells [41]. In contrast, CT26 tumors are immunologically hot, with a higher mutational load and higher frequencies of NK cells, DCs, and macrophages compared to A20 tumors, and are therefore anticipated to be more sensitive to immunotherapy [41].

Concomitant CV8102 and systemic anti-PD-1 treatment significantly improved anti-tumoral responses in the CT26 model, with long-lasting and systemic immune responses that controlled tumor re-challenge and non-injected distal tumors. Anti-PD-1 antibodies have been demonstrated to enhance anti-tumoral T cell responses in various settings by blocking the interaction between the T cell inhibitory receptor PD-1 and its ligand PD-L1 [42]. This effect is observed with anti-PD-1 antibodies alone and in combination with short double-stranded RNAs with 5’ppp that activate RIG-I [43], as was seen in this study. Based on our in vitro data, we hypothesize that CV8102-induced upregulation of PD-L1 on the surface of CT26 cells could be one explanation for the apparent synergy of CV8102 and anti-PD-1 antibodies in this study. Whether this also occurs in vivo remains to be determined. Since loss of PD-L1 on CT26 cells leads to increased T cell activation and tumor rejection [44], upregulation of PD-L1 could conversely hamper T cell activation and promote tumor growth, a mechanism that could be counteracted by PD-1 inhibition.

Transcriptome analysis of tumors demonstrated that treatment with CV8102 as monotherapy induces anti-viral and related immune responses driven by type I interferons, which are further enhanced and broadened by the addition of systemic anti-PD-1 treatment. By comparison, PD-1 blockade alone had little detectable effect on the tumor transcriptome, although it is important to note that the contribution of small cell populations such as PD-1-responsive infiltrating lymphocytes can be obscured in bulk RNA-Seq data where most of the RNA is derived from tumor and stroma cells.

Analysis on protein level confirmed the detected gene signatures and showed that increased concentrations of IFN-α, IFN-β, IL-6, and CCL5 were observed in tumors following treatment with CV8102, with and without PD-1 inhibition, compared with the buffer treatment group. This finding suggests that these cytokines are predominantly induced by CV8102, which is in line with the in vitro release of these cytokines by CV8102-stimulated murine CT26 tumor cells and human PBMCs [32, 34].

These data indicate that CV8102 could promote a pro-inflammatory environment by inducing tumor cells to release type I IFNs and chemoattractants, such as CXCL10, resulting in upregulation of PD-L1 and MHC-I. However, the increases in intratumoral cytokines resulted in only limited increases in serum concentrations, which is reflective of the good safety profile that has been observed for CV8102 in clinical studies [35, 36].

Assessments of immune cell infiltration to both tumor site and dLNs demonstrated significant increases of monocytes, NK cells and neutrophils within 14 h of treatment with a combination of CV8102 and anti-PD-1 antibodies. By 72 h post combined treatment, significantly higher levels of leukocytes and CD8^+^ T cells were observed in both tumors and dLNs compared with buffer only treatment. While concomitant CV8102 and PD-1 blockade treatment did not significantly increase cytokine production in the tumor environment or serum compared with CV8102 treatment alone, significant differences in immune cell activation at both, the tumor site and dLNs were observed. Interestingly, concomitant CV8102 and anti-PD-1 treatment did not lead to an increase in the Treg population in the tumor tissue, which was observed after anti-PD-1 treatment alone. However, it is unknown whether anti-PD-1 treatment increased the Treg population in the tumor by inducing cell infiltration or local differentiation/expansion, and how CV8102 interfered with this process.

In summary, our results demonstrate that CV8102 treatment is a promising approach for local cancer immunotherapy, especially to boost the anti-tumoral effect of systemic CPI therapies. Based on these observations, a clinical trial of CV8102, as monotherapy and in combination with immune checkpoint inhibition, is currently ongoing in patients with advanced melanoma, squamous cell carcinoma, skin or head and neck, and adenoid cystic carcinoma (ClinicalTrials.gov identifier: NCT03291002).

## Supplementary Information

Below is the link to the electronic supplementary material.

Supplementary file1 (PDF 645 kb)

## Data Availability

Relevant data that support these findings will be made available to qualified researchers upon request to the corresponding author.
