# Supplementary material for: Local immunotherapy with the RNA-based immune stimulator CV8102 induces substantial anti-tumor responses and enhances checkpoint inhibitor activity
Source: Cancer Immunol Immunother. 2022 Nov 2;72(5):1075–87. doi: 10.1007/s00262-022-03311-4 (PMC10110722; doi:10.1007/s00262-022-03311-4)
Supplement: Supplementary file 1 — Supplementary file1 (PDF 645 kb) [file 262_2022_3311_MOESM1_ESM.pdf]

Supplemental Material

Table S1 Intracellular and cell-surface staining reagents used for flow cytometry

Antigen	Flurochrome	Clone	Company	Staining
CD103	PE-DAzzle	2E7	Biolegend	Surface
CD107a	BV421	1D4B	Biolegend	Surface
CD11b	PE	M1/70	BD	Surface
CD11c	PE-CY7	PE-Cy7	Biolegend	Surface
CD19	BV421	6D5	Biolegend	Surface
CD206	BV711	C068C2	Biolegend	Surface
CD25	BV605	PC61	Biolegend	Surface
CD4	FITC	RM4-5	eBioscience	Surface
CD44	BV605	IM7	Biolegend	Surface
CD45	AF-700	30-F11	Biolegend	Surface
CD62L	APC	MEL-14	Biolegend	Surface
CD69	PE	H1.2F3	BD	Surface
CD8	PE-Cy7	53-6.7	eBioscience	Surface
CD80	BV605	16-10A1	Biolegend	Surface
CTLA-4	PerCP-Cy5.5	UC10-4B9	Biolegend	Intracellular
DX5 (CD49b)	PerCpCy5.5	HMα2	Biolegend	Surface
F4/80	APC	BM8	Biolegend	Surface
FOXP3	PE	FJK-16s	eBioscience	Intracellular
IFNg	BV711	XMG1.2	BD	Intracellular
LAG3	PE-Dazzle	C9B7W	Biolegend	Surface
Ly6C	FITC	HK1.4	Biolegend	Surface
Ly6G	PerCp-Cy5.5	1A8	Biolegend	Surface
MHCII	APC-Cy7	M5/114.15.2	Biolegend	Surface
Nkp46	APC	29A1.4	Biolegend	Surface
PD-1	BV711	29F.1A12	Biolegend	Surface
PD-L1	BV421	10F.9G2	Biolegend	Surface
TCRβ	BV650	H57-597	BD	Surface
TNF	PE-Dazzle	MP6-XT22	Biolegend	Intracellular

Supplemental Figures

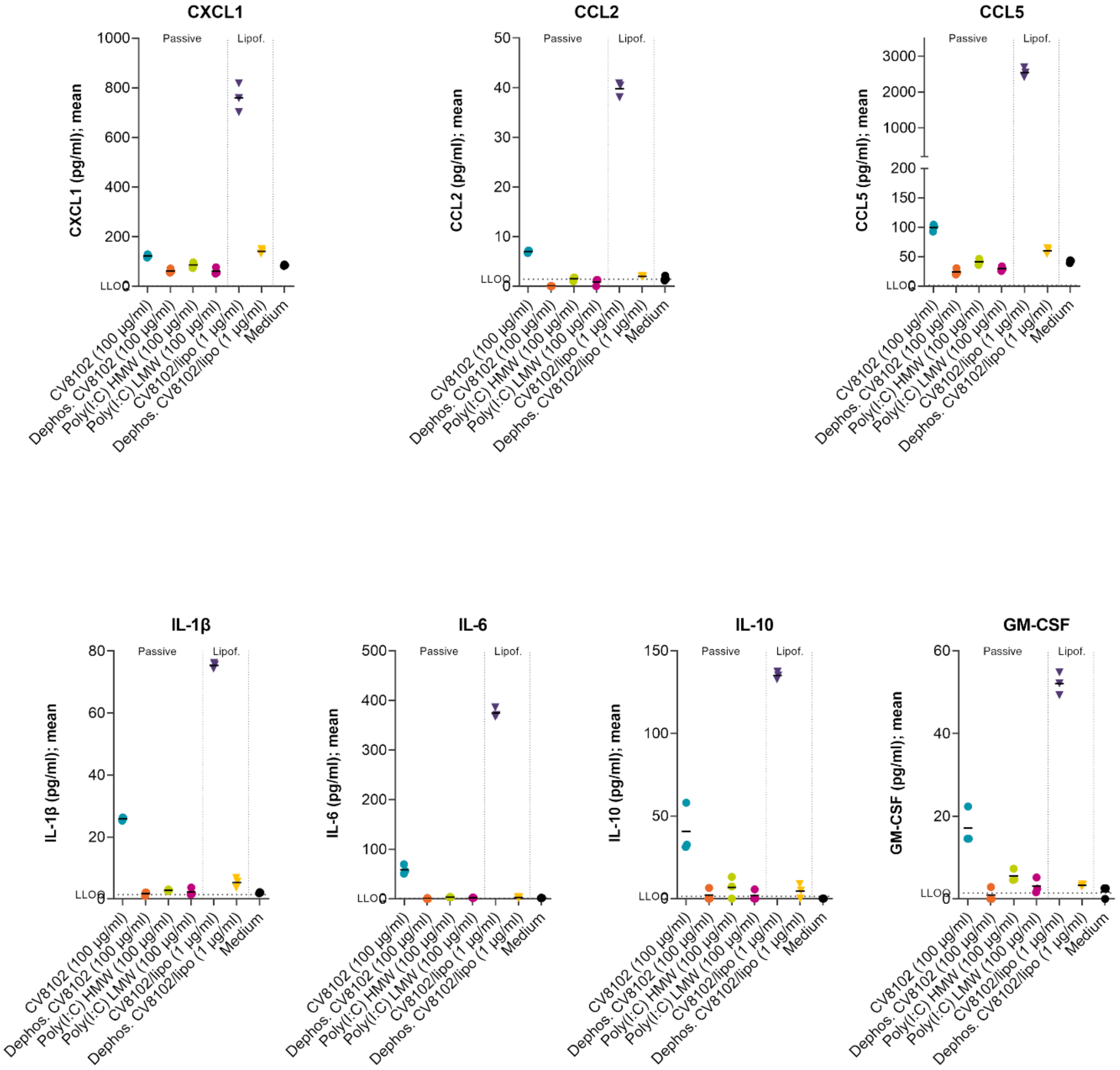

Fig. S1 CV8102 induces cytokine release in tumor cells *in vitro*

Additional cytokines measured in the supernatant of CT26 cells incubated with CV8102, dephosphorylated CV8102, and HMW or LMW poly(I:C) or transfected with CV8102 and dephosphorylated CV8102. See Fig. 1 for details.

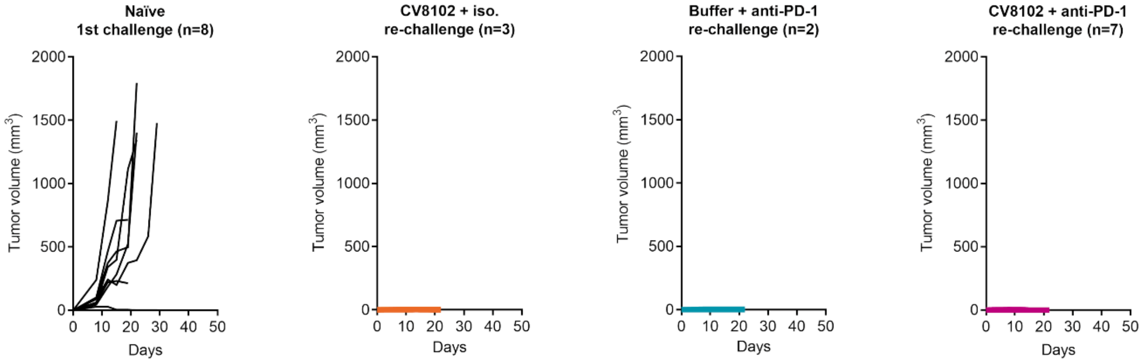

Fig. S2 Intratumoral CV8102 or systemic anti-PD-1 treatment protects against tumor rechallenge

Mice (n=2–7/group) with complete response to the primary CT26 challenge (see Fig. 4c) were re-challenged 16 weeks after the primary CT26 challenge with the same cells in the opposite flank. Tumor growth in individual mice is shown. Naïve mice (first challenge) served as controls. None of the animals received treatment. For re-challenged groups the initial treatment is indicated.

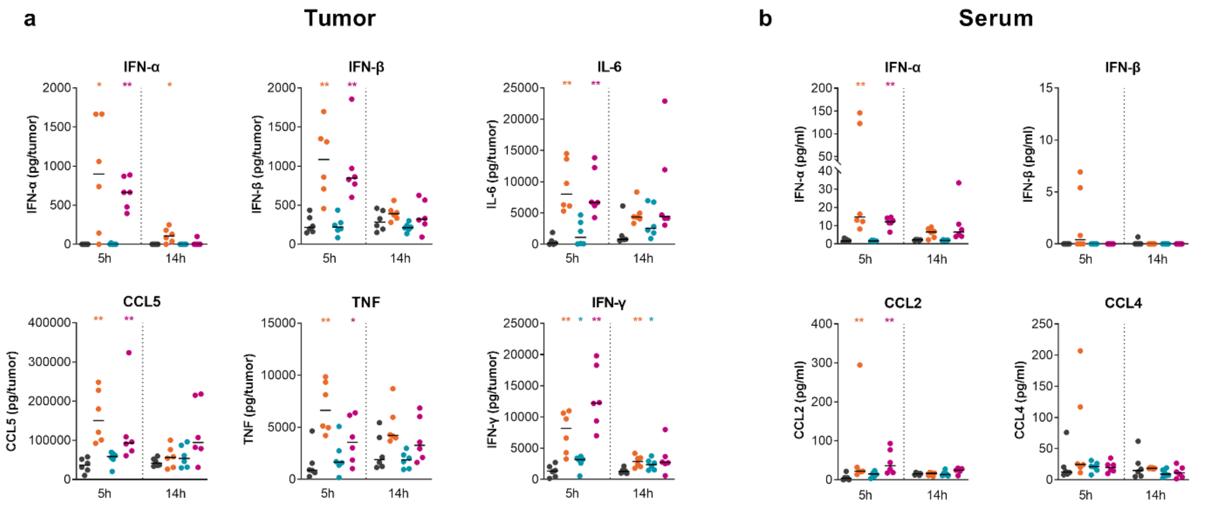

Fig. S3 Repeated intratumoral application of CV8102 induces cytokine release

Mice (n=6/group) were challenged on one flank with CT26 tumor cells on Day 0 and treated on Days 9, 13, and 16 with 100 µg CV8102 (i.t.) and 200 µg anti-PD-1 antibodies (i.p.) either alone or in combination; control animals received buffer (i.t.). Tumors and sera were collected 5 hours and 14 hours after the last treatment, and cytokine concentrations were determined in tumor lysates (**a**) and sera (**b**). Statistical analysis by Mann-Whitney test. All treatments are compared with buffer controls and significant changes are shown (*p<0.05, **p<0.01).
